# Real-time pressure mapping smart insole system based on a controllable vertical pore dielectric layer

**DOI:** 10.1038/s41378-020-0171-1

**Published:** 2020-08-10

**Authors:** Juan Tao, Ming Dong, Li Li, Chunfeng Wang, Jing Li, Yue Liu, Rongrong Bao, Caofeng Pan

**Affiliations:** 1 CAS Center for Excellence in Nanoscience, Beijing Key Laboratory of Micro-nano Energy and Sensor, Beijing Institute of Nanoenergy and Nanosystems, Chinese Academy of Sciences, 100083 Beijing, People’s Republic of China; 2 College of Optoelectronic Engineering, Shenzhen University, 518060 Shenzhen, People’s Republic of China; 3 Beijing Institute of Tracking and Telecommunications Technology, 100094 Beijing, People’s Republic of China; 4 Center on Nanoenergy Research, School of Physical Science and Technology, Guangxi University, Nanning, Guangxi 530004 People’s Republic of China; 5 School of Nanoscience and Technology, University of Chinese Academy of Sciences, 100049 Beijing, People’s Republic of China

**Keywords:** Sensors, Electrical and electronic engineering

## Abstract

Real-time monitoring of plantar pressure has significant applications in wearable biosensors, sports injury detection, and early diagnostics. Herein, an all-in-one insole composed of 24 capacitive pressure sensors (CPSs) with vertical pores in an elastic dielectric layer is fabricated by laser cutting. Optimized CPSs with a hexagonal configuration and a pore size of 600 μm possess good linearity over a wide detection range of 0–200 kPa with a sensitivity of 12 × 10^–3^ kPa^−1^. Then, a smart system including the all-in-one insole with the 24 CPS array, a data acquisition system with a wireless transmitter and a PC terminal with a wireless receiver is established for real-time monitoring to realize static and dynamic plantar pressure mapping. Based on this smart insole system, various standing and yoga postures can be distinguished, and variations in the center of gravity during walking can be recognized. This intelligent insole system provides great feasible supervision for health surveillance, injury prevention, and athlete training.

## Health: Another step forward for wearable sensors

A smart insole capable of monitoring a person’s gait could see use in wearable medicine, injury detection, or help to restore a healthy posture. Smart technologies capable of measuring physiological signals can help to monitor and improve human health. Recently, devices capable of mapping plantar pressure and distribution are seeing increasing use as predictors of a variety of health-related issues, such as exhausted running or walking, risk of slipping, or for detecting senile dementia. Led by Rongrong Bao from the Chinese Academy of Sciences, a team of researchers has developed an insole that uses an array of capacitive pressure sensors to monitor real-time data from static postures, such as standing or yoga poses, or dynamic activities like walking. The insole could see use as a wearable biosensor, in sports injury detection, or for diagnosing diseases.

## Introduction

With the prosperous development of the social economy, the demand for smart systems that can monitor various human physiological signals is increasingly vital for the early diagnosis or prevention of disease^1–5^. Often, smart devices focused on health surveillance can be utilized in many practical applications by monitoring physiological parameter information in a noninvasive way^2,6,7^, such as long-term accurate pulse monitoring using the radial artery for supervising the heart^8,9^, real-time nonjoint^10^ or joint^11,12^ movement monitoring of the body for revealing conditions of the muscle or skeleton, and intracranial pressure and temperature monitoring for treatment of traumatic brain injury^13^. At the same time, gait recognition on the basis of the plantar pressure distribution is also an essential aspect for health monitoring and has gradually become a concern^14–16^. Available plantar pressure mapping containing the amplitude and distribution of the pressure could be used as a reliable predictor or to warn of a variety of health-related issues, such as exhausted running or walking and the risk of slipping or senile dementia^17–19^.

To accomplish the various types of health monitoring mentioned above, tactile sensors acting as sensing modules are a vital part of smart systems and have attracted intensive attention^20–22^. Generally, elementary transmission mechanisms, including capacitance^23–25^, piezoresistivity^26–28^, and piezoelectricity^29–33^, are often utilized to convert external pressure into electrical signals. Compared with other transmission mechanisms, capacitive pressure sensors (CPSs) possess the advantages of a simple structure, low power consumption, and easy large-area fabrication^34^. Furthermore, numerous efforts have been made to enhance their fundamental performance, such as achieving a high sensitivity, superior stability, a wide detection range, and good linearity. Particularly, to enhance the sensitivity of capacitive sensors, pyramidal structures^35,36^, micropores^37–39^, and air gaps^40^ were fabricated in the dielectric layer to obtain a suitable Young modulus or Ag nanowires^41,42^ were embedded to properly increase the permittivity. A porous structure is commonly used in these methods to enhance the sensitivity. Additionally, it is difficult to simultaneously endow the sensor with high sensitivity, a wide detection range, and good linearity; thus, it is significant to compromise on these properties to obtain a better application effect in certain cases.

In this work, a smart insole system based on a CPS array with a vertical pore elastic dielectric layer is constructed to monitor static and dynamic plantar pressure mapping. First, different configurations and pore sizes are investigated to optimize the characteristics of the CPSs by a simple and effective method using laser cutting technology. The CPS with a hexagonal configuration and a vertical pore size of 600 μm is an adaptive and optimal choice, with a sensitivity of 12 × 10^−3^ kPa^−1^ over a wide detection range of 0–200 kPa, and has good linearity and excellent stability over 15,000 cycles. Then, a smart insole system including an all-in-one insole fabricated by laser cutting with a 24 CPS array, a data acquisition (DAQ) system with data reading of 28 groups of sensor array data per second and a wireless transmitter, and a PC terminal with a real-time digital and hotspot display and a wireless receiver is established. Based on this smart insole system, static and dynamic motions, including different standing postures, yoga asana, walking straight, turning around, falling down, and going upstairs, are investigated. The results show that this intelligent insole system can monitor and distinguish different plantar pressure mappings in real time, providing potential applications in wearable medicine, sports injury detection, athlete training, sports equipment design, and so on.

## Results and discussion

### Fabrication using laser cutting and structure of the all-in-one insole

**Fig. 1:**
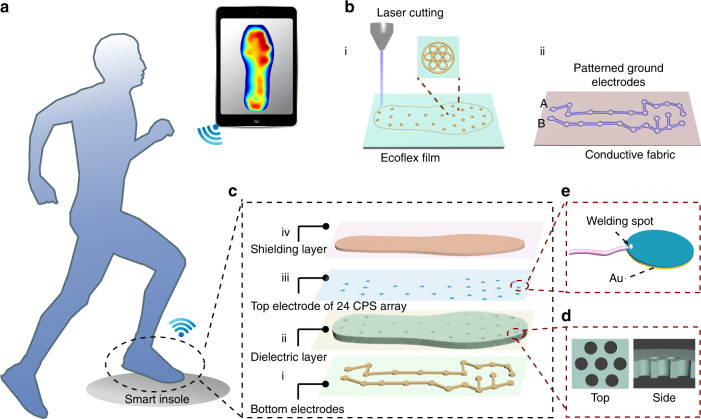
Schematic illustration of the smart wearable insole system. **a** Smart insole equipped on a pair of shoes to monitor the plantar pressure. **b** Diagram of the dielectric layer and bottom electrodes fabricated by laser cutting: i, an Ecoflex rubber film used as the dielectric layer; ii, a conductive fabric used as bottom electrodes, which are patterned into A for S1–S12 and B for S13–S24. **c** Basic structure of the insole consisting of four layers: i, bottom electrodes connected to the ground; ii, elastic dielectric layer with a 24-channel structured capacitive dielectric layer; iii, top electrodes of the 24 capacitive sensors; and iv shielding layer connected to the ACS port. **d** Top and side view diagrams of the vertical pores. **e** Illustration of the flexible screen-printed top electrodes with a welding hole

An insole smart system is constructed for real-time monitoring to realize pressure mapping of the foot and includes three parts: an all-in-one insole with a 24-channel capacitive sensor array, a DAQ circuit with a wireless transmitter, and a PC terminal with a wireless receiver, as depicted in Fig. 1a (more specific details about the circuit working principle will be described in the subsequent section). As illustrated in Fig. 1c, the insole consists of four layers: bottom electrodes, a middle dielectric layer, top electrodes, and a shielding layer. An Ecoflex film with a thickness of ∼800 μm is chosen as the rubber dielectric layer due to its low Young’s modulus, and a conductive fabric acts as the bottom electrode layer because of its flexibility and comfort when combined with shoes. The middle insole-shaped dielectric layer with 24 porous CPSs and patterned bottom electrodes A and B is obtained by laser cutting technology, which is a simple and effective way to obtain controlled porosity, as illustrated in Fig. 1b. The bottom electrodes are grounded electrodes and connected to the ground port in the chip, which is used to eliminate interference from the ground when walking. Herein, vertical pores with a hexagonal configuration are fabricated in the dielectric layer, aiming to increase the sensitivity of the sensor due to the lower Young’s modulus. At the same time, this kind of pore structure can improve the comfort of the insole, with the corresponding top and side views displayed in Fig. 1d. For the top electrodes, flexible screen-printed electrodes with a welding hole are adopted, as shown in Fig. 1e. Compared with the conventional contact conductor of silver paste, the welding hole provides a steady connection pathway with wires, guaranteeing enduring function of the smart insole in practical applications. Additionally, the radius of the disk electrode is 5 mm. The top shielding layer is also made of an insole-shaped conductive fabric to prevent interference from the environment.

### Optimization and basic performance of the capacitive sensor

Different configurations and pore sizes of the elastic dielectric layer are investigated to optimize the characteristics of the CPSs. The sensitivity of a CPS can be defined as *S* = ((*C* − *C*_0_)/*C*_0_)/Δ*p*, where *p* is the external applied pressure and *C*_0_ and *C* are the capacitance values of the sensor without and with the external applied pressure. Therefore, the slope of the relative capacitance variation–pressure curve represents the pressure sensitivity. Supplementary Fig. S1 shows a comparison of the sensitivity for different configurations, including square, hexagonal, and octagonal, which are correspondingly represented by *n* = 5, 7, and 9 (*n* is the number of pores) with the same pore radius of 500 μm. The sensitivity is gradually promoted with increasing number of pores due to the enhanced porosity of the dielectric. Simultaneously considering the structural stability, the hexagonal configuration is adopted. Although the sensitivity of the octagonal configuration (*n* = 9) is higher than that of the hexagonal configuration (*n* = 7), the stability of the structure is inferior due to the narrow interval between pores. Thus, the hexagonal configuration simultaneously presents better sensitivity and stability. Then, hexagonal configurations with different pore sizes, including 300, 400, 500, and 600 μm, are explored. It is worth noting that in this configuration, the dielectric layer space between pores with large sizes (such as 700 μm) is very narrow. Thus, the insulated dielectric layer will be easily fractured and disconnected in the actual experiment, which would bring about contact of the top and bottom electrodes.

**Fig. 2:**
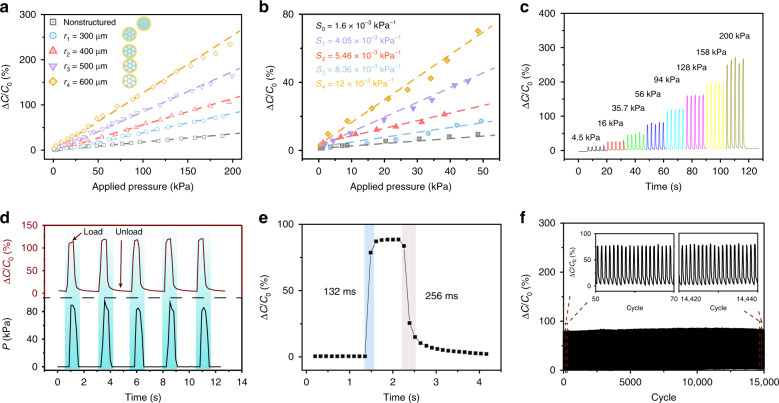
Basic performance of the prepared capacitive pressure sensors. **a** Capacitance variation–pressure curves for the hexagonal configuration with different pore sizes, including 300, 400, 500, and 600 μm, and the nonstructured configuration in the range of 0–200 kPa. **b** Amplification of the sensitivity curves in the range of 0–50 kPa. **c** Relative capacitance variation at different external pressures, including 4.5, 16, 35.7, 56, 94, 128, 158, and 200 kPa. **d** Relative capacitance variation and corresponding applied pressure–time curves at the same time. **e** Response times for rising and falling loads. **f** Fifteen thousand cycle repeatability of the sensor under an applied pressure of 50 kPa

As shown in Fig. 2a, compared with the nonstructured dielectric layer, the sensitivity of all the sensors with the pore structure is improved, and the pore size of 600 μm leads to the highest sensitivity of 12 × 10^−3^ kPa^−1^ over a wide detection range of 0–200 kPa, which is 7.5 times that of the nonstructured sensor. It is worth noting that the CPSs with vertical pores have a superior linearity over a wide detection range up to 200 kPa, laying a good foundation for the later planar pressure mapping. Figure 2b shows a comparison of the pressure response curves in the range of 0–50 kPa, which still maintain the same trends and results. Thus, the CPS of the hexagonal configuration with a 600 μm pore size is considered an optimal choice to perceive plantar pressure in this smart insole system. As displayed in Fig. 2c, the relative capacitance variation at different external pressures ranging from 4.5 to 200 kPa is presented for five trials, demonstrating the feasibility of perceiving different external pressures and a preferable stability at every pressure. Capacitance response curves are presented for gradually increasing and decreasing pressure (Supplementary Fig. S2a). Supplementary Fig. S2b shows good consistency in the range of 0–200 kPa during the increasing and decreasing pressure process, demonstrating the superior resilience and stability. Additionally, relative capacitance variation and corresponding applied pressure–time curves are exhibited (Fig. 2d), proving the real-time response to loading and unloading. Figure 2e shows that the measured response times for rising and falling loads are ~132 and 256 ms, respectively, which is restrained by the readout of seven points per second of the LCR meter measuring device and the speed of the linear motor. Finally, the 15,000 cycle repeatability of the sensor under an applied pressure of ~50 kPa is measured (Fig. 2f), demonstrating the excellent durability of the sensor. The inset figures illustrate amplifications of the initial 50–70 and final 14,420–14,440 cycles, where the capacitance variation values remain almost the same, proving the good stability. The superior characteristics of sensitivity, detection range, linearity, and stability guarantee the availability and feasibility in practical applications.

### Design and principle of the smart insole system

**Fig. 3:**
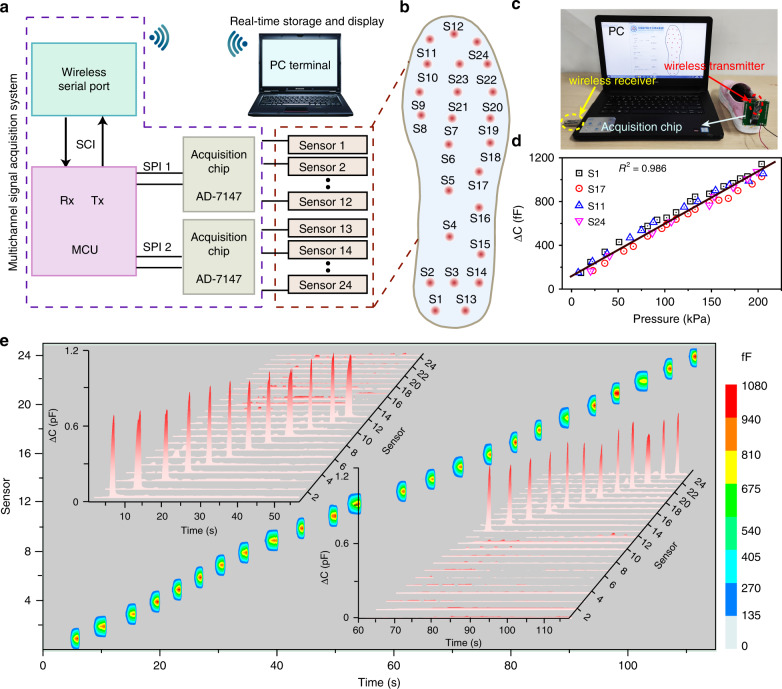
Fundamental design and principle of the smart insole system. **a** Principle circuit diagram of the smart insole system including three parts: insole with the 24-channel capacitive pressure sensor array, DAQ circuit module with the wireless transmitter, and PC terminal with the wireless receiver. **b** Configuration of the distribution of 24 sensors on the insole, named S1 to S24: S1–S12 distributed on the left connected to chip 1, and S13–S24 distributed on the right connected to chip 2. **c** Photograph of the smart insole system. **d** Capacitance variation–pressure revision curve of the insole for the system. **e** Capacitance–time response curves of 24 sensors obtained by using a similar force in sequence from S1 to S24

The smart insole system mainly comprises a 24-channel CPS array, a DAQ circuit with a wireless transmitter, and a PC terminal with a wireless receiver, with the circuit diagram depicted in Fig. 3a. First, for the 24-channel CPS array (red dashed box), the sensors are distributed in the insole according to the configuration in Fig. 3b. Five, seven, and twelve sensors are arranged in the heel, middle, and forefoot region, with sensors 1–12 and 13–24 distributed in the left and right parts of the insole, respectively. Bottom electrodes A and B correspond to S1–S12 and S13–S24.

For the DAQ circuit module (purple dashed box), the AD7147 chip is adopted, which is a programmable capacitance digital converter from Analog Device Company of America. The resolution of the chip is ~1 fF. It has 12-channel capacitance signal input. The on-chip autocalibration logic can automatically compensate for environmental changes and perform self-adaptive threshold adjustment. The data transmission adopts a serial peripheral interface (SPI)-compatible synchronous serial interface. The working voltage is ~2.6 to 3.3 V and the working current is low (full power mode <1 mA). There are two AD7147 chips in the system because there are 24 capacitance sensors in this array. PIC16F1526 is a mid-range MCU of an American company. It has two SPI synchronous serial interfaces, two AD7147 and two serial communications interface asynchronous serial interfaces, which can be used for upper computer communication. To facilitate communication with the upper computer, the wireless serial port and a battery power supply are used to avoid cable connection. The collection speed of the whole system is ~28 groups of sensor array data per second, which is sufficient to realize collection of plantar pressure distribution data during human motion.

The PC terminal is used to store and display the data of the sensor array in real time, with the display mode divided into digital and hotspot display. At the same time, a thermodynamic diagram display mode has also been developed using cubic B-spline surface fitting technology. A photograph of the smart system is exhibited in Fig. 3c, including the smart insole in shoes, acquisition chip, wireless transmitter and receiver, and PC terminal. To eliminate interferences caused by this whole system, a system revision measurement is carried out. Any four sensors, including S1 in the heel part, S17 in the middle part, and S11 and S24 in the forefoot of the insole, can be chosen to measure the capacitance variation (Δ*C*) under different pressures. It can be observed that the scatter of the capacitance–pressure data is approximately uniform and still maintains a linear trend in the range of 0–200 kPa (Fig. 3d). A capacitance variation–pressure revision curve for this smart insole system is simulated. Thus, subsequent plantar pressure measurements are subjected to this simulated curve to make them closer to the actual pressure values. Finally, it is necessary to ensure that every channel of the sensors will not generate mutual crosstalk and can function independently. Hence, the 24 channels are tested individually under a similar force in sequence from S1 to S24, as seen in Supplementary Video S1. It can be observed that the capacitance–time response curves almost maintain the same capacitance variation amplitude of ~900 fF, and every sensor can function well independently with no interference from the other sensors, as seen in Fig. 3e.

### Static plantar pressure mapping based on the smart insole system

**Fig. 4:**
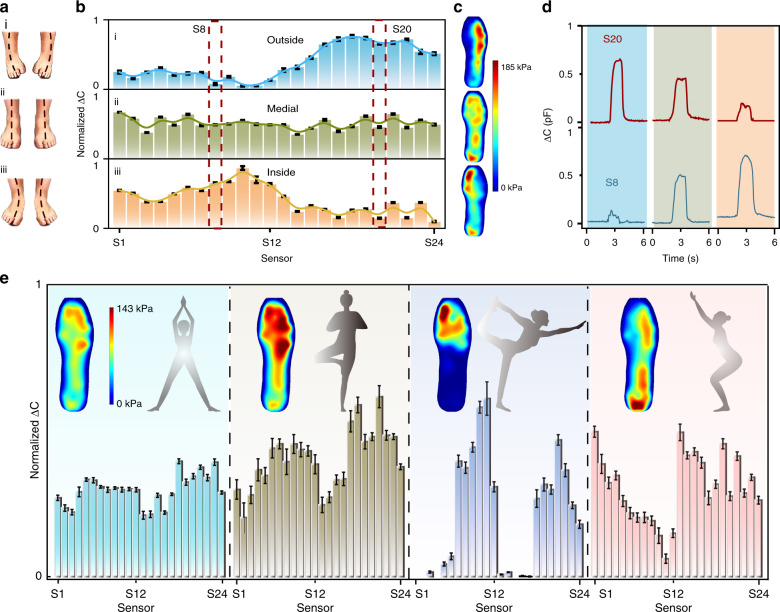
Illustration of static plantar pressure mapping based on the insole system. **a** Three standing postures corresponding to underpronation (i), normal posture (ii), and overpronation (iii). **b** Capacitance variation (Δ*C*) histogram of the 24-channel sensors in these three standing postures for the right foot: i, underpronation; ii, normal posture; iii, overpronation. **c** Thermodynamic diagram of the plantar pressure for the three standing postures. **d** Capacitance response curves of S8 (left part) and S20 (right part) of the right foot. **e** Δ*C* histogram of the 24 sensors, diagram of the thermodynamic pressure, and four kinds of yoga posture

Based on the prepared smart insole system, static plantar pressure mapping is investigated. It is significant and essential to be aware of the standing posture, which is of concern for quality of life and conducive to redressing unhealthy posture. Hence, the experimenter’s right foot wearing a shoe loaded with the smart insole system is posed in three ways corresponding to underpronation (i), normal posture (ii), and overpronation (iii), as shown in Fig. 4a. The capacitance variation (Δ*C*) of the 24 sensors on the right foot for these three standing poses is shown in Fig. 4b. The tendency of the medial type is almost a straight line (green line), demonstrating the relatively uniform pressure distribution over the whole right foot. However, for the outside and inside types, the Δ*C* values of S1*-*S24 present two contradictory trends, with the S1–S12 values lower than the S13–S24 values and the S1–S12 values higher than the S13–S24 values, respectively. According to this result, it can be deduced that the pressure distribution over the left part (inside) of the right foot is lower than that over the right part (outside) for the underpronation type, while the pressure distribution over the right part (outside) is lower than that over the left part (inside) for the overpronation type. In accordance, the thermodynamic diagrams of the three standing postures are also displayed in Fig. 4c, indicating the change in the center of gravity of the whole body. Additionally, S8 of the left part and S20 of the right part are chosen for comparison, and the Δ*C*–*t* curves of the three types are displayed in Fig. 4d. Identical evidence can be observed in that the actual capacitance variation amplitudes of S8 show lower, similar and higher trends compared with S20 for underpronation, normal posture and overpronation, respectively. These results indicate that this approach is effective for realizing plantar static pressure mapping of a standing posture, which can provide valid data for adjusting the standing posture to a normal and healthy position or for choosing suitable shoes.

Additionally, this smart insole system is also appropriate for monitoring the static pressure distribution during yoga, which is an exercise integrating physical, mental, and spiritual training. Thus, as displayed in Fig. 4e, four different kinds of yoga posture, which can be expressed as standing on both feet, standing on the right foot, stretching on one’s forefoot, and squatting, are performed by the experimenter, with the corresponding Δ*C* histograms of the 24 sensors, thermodynamic pressure mapping, and diagram of the posture exhibited. Compared with standing on both feet, the amplitude and error bar of the Δ*C* histograms of the 24 sensors for standing on the right foot are higher and larger, indicating that the right foot is suffering from greater pressure and instability of the center of gravity of the human body. At the same time, the average pressure for the single-foot standing is approximately two times that for both feet standing according to the thermodynamic pressure diagram. It is worth noting that the pressure distribution on the heel is relatively low compared to that on the forefoot, mainly because the body of the experimenter is unstable and the experimenter leans forward. For stretching on one’s forefoot, the pressure distribution is mainly loaded in the forefoot and seldom loaded on the back heel, which is nearly separated from the shoes in this posture. Compared with stretching on one’s forefoot, the center of gravity in the squatting action is inclined toward the heel, with the thermodynamic diagram and Δ*C* histogram both illustrating this. In this posture, pressure distribution still occurs on the forefoot despite the center of gravity being rearward because there is intimate contact between the feet and shoes.

### Dynamic plantar pressure mapping based on the smart insole system

**Fig. 5:**
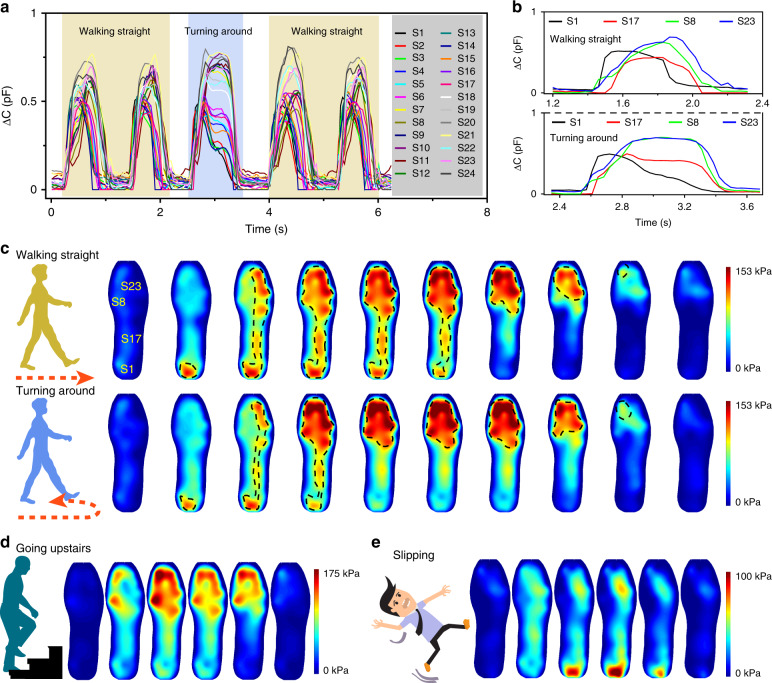
Diagram of dynamic plantar pressure mapping based on the insole system. **a** Capacitance response curves of the 24-channel sensors under the conditions of walking straight and turning around by the experimenter. **b** Capacitance response curves of S1 in the heel, S17 in the middle foot, and S23 and S8 in the forefoot in the case of walking straight and turning around. **c**, **d**, **e** Dynamic pressure mapping for walking straight and turning around, slipping, and going upstairs, respectively

To realize the diagnosis or prevention of certain diseases, gait recognition is necessary. Thus, dynamic plantar pressure mapping based on the smart insole system is researched. Figure 5a illustrates the capacitance response curves of the 24-channel sensors when the experimenter walks, and it can be observed that every cycle of the curve corresponds to one pace. Twenty-eight groups of sensor array data per second are recorded in this system, and every group containing 24 points can be a piece of a plantar pressure mapping. As seen in Supplementary Video S2, the dynamic plantar pressure mapping composed of all pressure photographs accords with the walking pace of the right foot. According to the video, the variation in the center of gravity of the human body while walking can be obviously observed at every pace, indicating achievable real-time display of our plantar dynamic pressure mapping based on this intelligent insole system.

As depicted in Fig. 5b, S1 in the heel, S17 in the middle foot, and S23 and S8 in the forefoot are chosen to analyze the gait when going straight and turning around. The heel, middle of the foot, and forefoot land on the ground in the first half, but are off the ground in the latter half of the space in sequence; in accordance, the capacitance peak values of S1, S17, and S23 appear in order, as depicted in Fig. 5b. Simultaneously, in line with the abovementioned results, plantar pressure mapping of the whole process of one pace when walking straight and turning around is displayed in Fig. 5c. Similarly, the results all indicate regular variations in the center of gravity from the heel to the middle of the foot to the forefoot, corresponding to the aforementioned curves. Compared with going straight, the pressure distribution on the forefoot is larger when turning around, which can be observed in the thermodynamic pressure diagram when turning around. The presence of a larger pressure distribution on forefoot is mainly attributed to the existence of a twist of our feet when turning around. In addition, when the experimenter is going upstairs, the corresponding pressure mappings are illustrated in Fig. 5d, which shows that the center of gravity is mainly loaded on the forefoot of the right foot in a whole process of going upstairs. A slip and fall action is simulated, with the corresponding dynamic pressure mapping illustrated in Fig. 5e. It can be observed that the center of gravity changes from the forefoot to the heel, indicating an imbalance of the body. In this case, our smart insole system can provide significant and effective follow-up or enable precautions to be taken to reduce the risk of injury. Timely help and rescue is of great significance to avoid further injury and can be provided since dynamic pressure mapping can be realized in real time. Furthermore, when this mapping is combined with machine learning by leveraging large-scale datasets collected based on the smart insole system, an advanced prediction or warning alert could be generated to prevent falling down once a person is estimated to be in relatively dangerous circumstances. Thus, the real-time pressure monitoring based on this smart insole system could be helpful for timely rescue or warning to prevent a person from falling down, providing great potential applications in artificial intelligence and the internet of things. In summary, this smart system can monitor the dynamic pressure mappings and distinguish our gait in walking, turning around, going upstairs, falling down, and so on, providing potential for early diagnosis or prevention of disease.

## Conclusion

A facile intelligent insole system based on a capacitive sensor array is created in this work to monitor the plantar pressure distribution. First, vertical pores in the middle elastic dielectric layer are fabricated by laser cutting, which is a simple and effective method. Different configurations consisting of squares, hexagons, and octagons and pore sizes of 300, 400, 500, and 600 μm are investigated to optimize the performance of the sensor. Hexagonal configuration with a 600 μm pore size is chosen as the optimal option due to the high sensitivity. The basic performance of the CPS lies in the relatively high sensitivity of 12 × 10^−3^ kPa^−1^ over a wide detection range of 0–200 kPa, good linearity and excellent stability. Then, an all-in-one insole, composed of four layers consisting of patterned bottom electrodes, a rubber dielectric layer with a 24 CPS array, 24 flexible top electrodes, and a shielding layer, is fabricated by laser cutting. Furthermore, a smart system including the all-in-one insole with the 24 CPS array, a DAQ circuit with a wireless transmitter, and a PC terminal with a wireless receiver is established. Based on this smart insole system, real-time static and dynamic plantar pressure mappings are monitored, including during various motions, such as different standing postures, yoga asana, walking straight, turning around, falling down, and going upstairs. The results show that this intelligent insole system can monitor and distinguish different standing postures, which could provide an indication to help restore a healthy and normal posture. This system can also enhance the technique for yoga trainers since different yoga postures can be monitored. Even during dynamic motion of a person, this system can simulate the change in the center of gravity while walking straight or turning around. In addition, the action of falling down can be monitored in real time, which could be useful for timely rescue or warning to prevent falling down, especially for elderly people. Hence, this smart insole system for real-time monitoring to realize plantar pressure mapping is of great significance, providing potential applications in wearable medicine, sports injury detection, athlete training, and sports equipment design.

## Materials and methods

### Preparation of capacitive sensors with different configurations and pore sizes

The sandwich structure of a CPS contains two flexible electrodes and an elastic dielectric layer. The middle elastic dielectric layer is made of Ecoflex (0010) rubber. First, 2 g of Ecoflex silicone elastomer (Smooth-On 00-10, mixing part A and part B in a ratio of 1:1 by weight) is poured into a 3d groove (3 × 6 × 1 cm) made of acrylic sheet and degassed in vacuum for 5 min to remove the bubbles at room temperature. The 3d groove with Ecoflex is left standing for 2 h to obtain a flat surface and a uniform thickness of ~800 μm. Then, the Ecoflex film stripped from the 3d groove is used after drying for punching by laser cutting. According to the pattern prepared by Auto CAD, including different square, hexagonal, and octagonal configurations with the same pore radius of 500 μm and hexagonal configurations with pore radiuses of 300, 400, 500, and 600 μm, capacitive sensors are obtained by laser cutting with appropriate power and speed, with an effective area of a disk of 5 mm. Then, flexible printed electrodes with a disk radius of 5 mm are adopted as the top and bottom electrodes, with a welding hole connected to conductive wire. Finally, the sensor is obtained as a sandwich structure with Ecoflex in the middle and flexible printed electrodes on the top and bottom face to face.

### Measurement of prepared capacitive sensors

The force (external pressure) applied on a capacitive sensor is measured by a force gauge (HANDPI, HP-50). Different forces are realized by a linear motor. The capacitance variation of the sensor is measured by using an Agilent (E4980A) Precision LCR meter at a frequency of 80 kHz and a voltage of 1 V. The experimental data of a single sensor are collected by a customized Lab VIEW program.

### Fabrication of the all-in-one insole

An Ecoflex film (15 × 30 cm^2^) with a thickness of 800 μm is obtained by the aforementioned method. Then, using laser cutting technology on the Ecoflex film, an insole-shaped middle dielectric layer with the corresponding structured CPS array is obtained. Then, conductive fabric with the desired thickness purchased from the internet is also patterned into bottom electrodes A (designed by connecting S1 with S12 on the left part) and B (designed by connecting S13 with S24 on the right part) by laser cutting technology. Then, 24 flexible printed electrodes are soldered using a welding table to act as top electrodes. Then, a commercial silicone binder is used to assemble these three layers together via layer-by-layer processing, and a thin Ecoflex film is evenly coated on the top electrodes as an encapsulation layer by the spin-coating method. Finally, an insole-shaped conductive fabric fabricated by laser cutting is laminated on the topside.

### Measurement based on the intelligent insole system

The aforementioned all-in-one insole is soldered to a DAQ chip supplied with an external battery of 5 V, and a wireless transmitter and a wireless receiver are inserted into the DAQ port and PC port, respectively. Thus, a real-time monitoring insole system is established. In this way, the capacitance variation (Δ*C*) under different pressures for system revision is measured, where the applied force is caused by a human hand holding an iron nib with a radius of 5 mm (whose contact area corresponds to the effective electrode area), and a force meter (HANDPI, HP-50) is placed under the sensor to record the magnitude of the force. Data collection is performed in this system by the DAQ chip, with data reading of ~28 groups of sensor array data per second. Then, various motions are measured and recorded by this system for the experimenter wearing shoes with the insole system.

### Simulation of pressure mapping of the insole

There are only 24 discrete points of pressure sensor data in the intelligent insole system with an uneven distribution. To analyze the pressure distribution characteristics of the whole foot, we use the cubic B-spline surface interpolation method to interpolate the discrete pressure measurement values to the whole foot and realize continuity of the pressure distribution. The B-spline function has many advantages, such as convenient calculation, high precision, and high numerical stability.

## Supplementary information

Supplementary information for Real-time Pressure Mapping Smart Insole System Based on a Controllable Vertical Pore Dielectric Layer

Video S1. Response of 24 channel capacitive pressure sensors independently

Video S2. Dynamic pressure mapping during walking
