# Supplementary material for: Real-time pressure mapping smart insole system based on a controllable vertical pore dielectric layer
Source: Microsyst Nanoeng. 2020 Aug 10;6:62. doi: 10.1038/s41378-020-0171-1 (PMC8433384; doi:10.1038/s41378-020-0171-1)
Supplement: Supplementary file 1 — Supplementary information for Real-time Pressure Mapping Smart Insole System Based on a Controllable Vertical Pore Dielectric Layer [file 41378_2020_171_MOESM1_ESM.docx]

Supplementary information

**Real-time Pressure Mapping Smart Insole System Based on a Controllable Vertical Pore Dielectric Layer**

*Juan Tao, Ming Dong, Li Li, Chunfeng Wang, Jing Li, Yue Liu, Rongrong Bao* and Caofeng Pan**


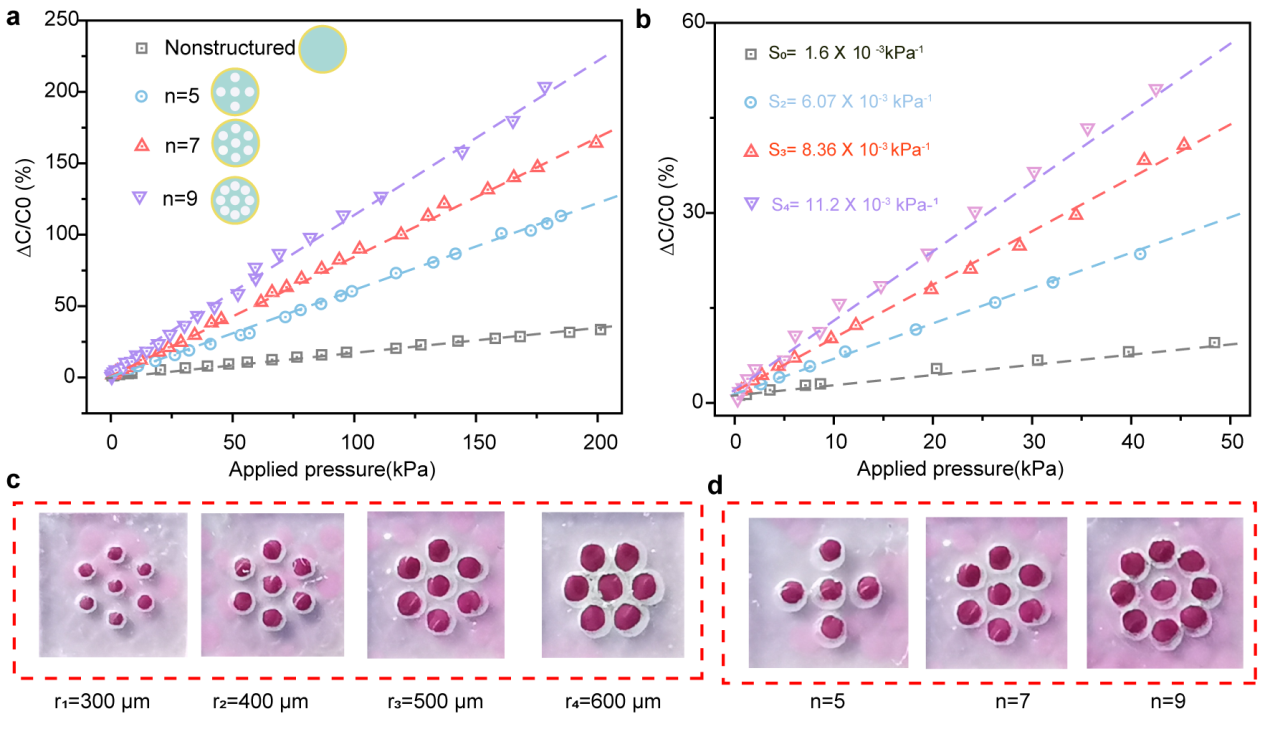


Fig. S1 **Sensitivity of different configuration including nonstructured, square, hexagon and octagon. a, b** Sensitivity comparison of different configuration including nonstructured, square (n=5), hexagon (n=7) and octagon (n=9) in the range of 0-200 kPa and 0- 50 kPa, respectively; **c, d** Optical photographs of different pore size including 300, 400, 500, 600 μm, and configuration of dielectric layer, respectively.

Fig. S1a illustrate the comparison of sensitivity for different configuration including nonstructured, square (n=5), hexagon (n=7), and octagon (n=9) with the pore size of 500 μm, where n is the number of pore in dielectric layer with an effective radius of 5 mm. It can be observed that these capacitance response curves remain a good linearity in a wide detect range of 0-200 kPa, and the sensitivity will be enhanced with the increased number of pores. That is because that increased number of pores will reduce the Young modulus and the dielectric layer is easy to be deformed. Further, amplification of 0-50 kPa is shown in Fig. S1b, compared with nonstructured sensor, the sensitivity is improved from 1.6 × 10^-3^ kPa^-1^ to 11.2 × 10^-3^ kPa^-1^. However, despite the sensitivity of the octagon (n=9) is higher than hexagon (n=7), the stability of structure is inferior due to the narrow interval between pores, shown in Fig. S1c. Optical photographs of different pore size and configuration of Ecoflex dielectric layer by laser cutting have been illustrated in Fig. S1c and Fig. S1d. It can be obviously observed that the pores are regular and controllable.


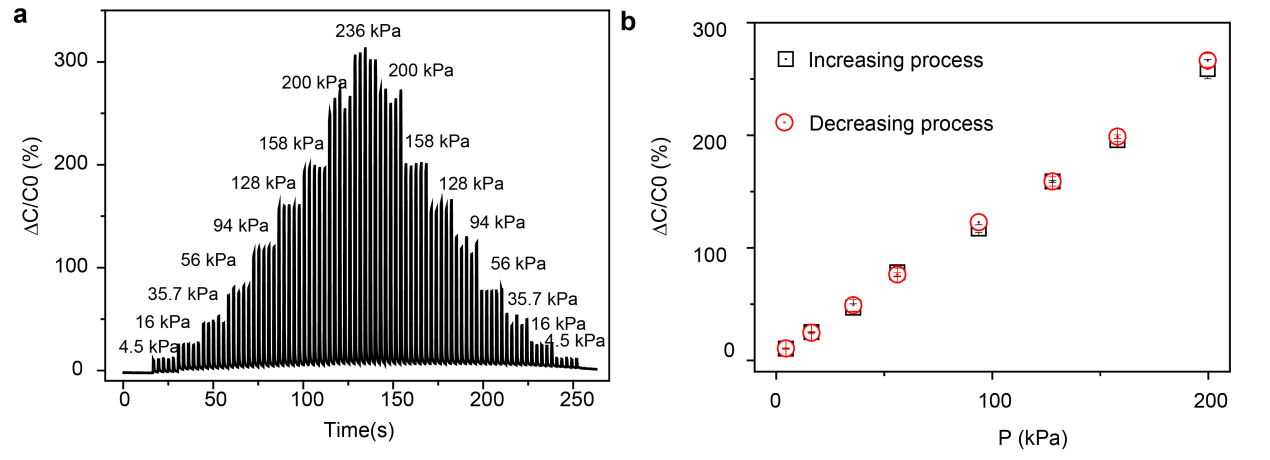


Fig. S2 **Resilience performance of sensors. a, b** Capacitance response-pressure curves and scatter diagram of CPS with hexagon and pore size of 600μm when increasing and decreasing applied force, respectively.

When applying different pressure with an increasing and decreasing process, the corresponding response curves with 5 cycles in every force and according scatter diagram are displayed in Fig. S2. The results show the sensors possess superior stability and resilience in different external applied pressure.

Video S1. Response of 24 channel capacitive pressure sensors independently

Video S2. Dynamic pressure mapping during walking
